# Galvanic vestibular stimulation for the postural rehabilitation of HTLV-1-associated myelopathy

**DOI:** 10.3389/fnhum.2024.1507559

**Published:** 2024-12-19

**Authors:** Tatiana Rocha Silva, Ludimila Labanca, Júlia Fonseca de Morais Caporali, Mauricio Campelo Tavares, Nathália de Castro Botini Rausse, Maria Júlia Amaral Abranches de Almeida, Maxmilliam de Souza Martins, Laura Fernandes Amorim, Léo Dantas Sitibaldi, Denise Utsch Gonçalves

**Affiliations:** ^1^Programa de Pós-Graduação em Infectologia e Medicina Tropical, Faculdade de Medicina da Universidade Federal de Minas Gerais, Belo Horizonte, Brazil; ^2^Programa de Pós-Graduação em Ciências Fonoaudiológicas, Faculdade de Medicina da Universidade Federal de Minas Gerais, Belo Horizonte, Brazil; ^3^Bolsista do CNPq na modalidade Produtividade em Desenvolvimento Tecnológico e Extensão Inovadora, Brasília, Brazil

**Keywords:** postural balance, vestibular diseases, central nervous system, electric stimulation, electric stimulation therapy

## Abstract

**Introduction:**

Galvanic vestibular stimulation (GVS) is a simple, safe, and noninvasive method of neurostimulation that can be used to improve body balance. Several central nervous system diseases cause alterations in body balance, including HTLV-1-associated myelopathy (HAM).

**Objective:**

To test GVS as a balance rehabilitation strategy for HAM.

**Methods:**

This study is a quasi-experimental clinical trial in which postural balance was compared before and after a GVS rehabilitation protocol applied to 20 patients with HAM, 12 women and 8 men, average age of 78 and 79 years, respectively. They were followed for nine months after the end of the GVS protocol, which consisted of one GVS session per week for 12 consecutive weeks. The GVS current intensity was progressively increased from 1.0 milliamperes (mA) to 3.5 mA until the third session and maintained at 3.5 mA until the 12th session. The electrical stimulation time progressively increased from 9 min in the first session to 18 min in the second session and maintained at 30 min from the third session onwards. Postural balance was assessed by Time up and go test (TUG), Berg balance scale (BBS) and posturography that were performed before the beginning of the intervention, during the intervention (6th week), at the end of the intervention (12th week) and after 9 months of follow-up without electrical stimulation.

**Results:**

In a blind comparison, in the 12th week of stimulation, improvement was observed in all the tests. In TUG, time in seconds changed from 28 before to 18 after GVS (*p* < 0,001). In BBS, the score changed from 29.00 before to 41.00 points after GVS. In posturography, the stability limit improved after the intervention (*p* < 0.05). However, after nine months without stimulation, the gain was lost for TUG, for BBS and for stability limit.

**Conclusion:**

GVS was an effective method to improve postural instability of patients with HAM in the short term, but the gain in postural stability was not maintained in the long term. A device for home use may be an option for long-term use.

## Introduction

The postural control system is robust enough to regulate balance in unstable conditions and versatile enough to allow a stable visual image during quick movements of the head. In the orthostatic position, the human body does not remain motionless but oscillates. Such oscillations with linear or angular body movements are neuromuscular responses to maintain postural balance (Chen et al., 2021). In case of sudden instability, the central nervous system (CNS) generates coordinated responses to maintain postural balance, and its integrity is necessary for the recognition of positions and movements of the head concerning the body and the environment (Mitsutake et al., 2022). To maintain proper postural balance, the CNS depends on afferent information from the proprioceptive, the vestibular and the visual systems that promote the interaction of the body with the space (Nguyen et al., 2022).

Galvanic Vestibular Stimulation (GVS) is a non-invasive method that stimulates the vestibular system, including vestibular sensors, neural pathways, vestibular nuclei, and cortical areas that receive integrated vestibular input (Pires et al., 2022).

GVS consists of a transcranial electrical current that polarizes the vestibular nerves, separating and accumulating positive and negative electrical charges in two distinct and opposite regions of the vestibular system. In a binaural and bipolar configuration, when applying electrical stimulation to both mastoid processes, vestibular afferents on one side are excited (cathode), and on the other are inhibited (anode), changing the resting potential (Chen et al., 2021). This process activates the central vestibular system and correlated synapsis (Kwan et al., 2019). In this way, GVS modulates posture and balance (Chen et al., 2021), oculomotor responses (Tohyama et al., 2021) and spatial orientation (Wuehr et al., 2023).

CNS interprets the dipole between the labyrinths generated by the GVS as a movement of the head (Carmona et al., 2011). Cathodic stimuli depolarize, while anodic stimuli hyperpolarize vestibular afferent fibers (Carmona et al., 2011). GVS generates a temporary oscillation of the body towards direct current stimulation generates a sensation of falling towards the cathode compensated by a slight inclination towards the anode (Fitzpatrick and Day, 1985). Oculomotor movement characterized by horizontal and torsional nystagmus toward the cathode is also observed (Nguyen et al., 2022). After stimulation, posture and ocular movements return to their original position.

GVS can be a great auxiliary tool in rehabilitating uncompensated peripheral vestibular diseases, neurodegenerative diseases and myelopathies that cause postural instability (Mitsutake et al., 2022; Nguyen et al., 2022). Electrical stimulation appears to promote increased spinal cord responses related to posture, resulting in improved balance, but further studies need to be carried out to confirm or deny this hypothesis. Rehabilitation with GVS is effective in terms of neuronal stimulation (Carmona et al., 2011). In addition, GVS is a low-cost and easy-to-perform method.

Several CNS diseases cause changes in body balance, and the available rehabilitation modalities have limited results regarding balance gain. An example associated with imbalance is HTLV-1 Associated Myelopathy (HAM) (Labanca et al., 2015).

HAM is a neurological disease characterized by spinal cord inflammation with damage medullary alterations occur mainly in of the entire neuroaxis that predominates in the thoracolumbar region (Labanca et al., 2015; Araujo and Silva, 2006; Silva et al., 2020; Silva et al., 2019; Labanca et al., 2018). The corticospinal pathway and, due to anatomical surroundings, the vestibulospinal pathways are affected, leading to postural instability (Fitzpatrick and Day, 1985; Labanca et al., 2015; Araujo and Silva, 2006; Silva et al., 2020; Silva et al., 2019). Due to the alteration of the vestibulospinal pathway, the HTLV-1-infected person can present a loss in body balance and complain of dizziness, which can precede the definite diagnosis of HAM (Labanca et al., 2015). Recent studies have shown changes in body balance based on posturographic assessment on a force platform in which individuals with HAM and asymptomatic carriers were compared (Patrício et al., 2020; Vasconcelos et al., 2019).

Given the challenge of rehabilitating postural instability due to a neurological disorder, the present study aimed to present a before and after GVS comparison of patients with postural instability related to HAM and their follow-up to assess the long-term effect of electrical stimulation.

## Methods

### Ethical aspects

This research was conducted in accordance with the principles expressed in the Declaration of Helsinki and was approved by the Research Ethics Committee from Universidade Federal de Minas Gerais (COEP UFMG), logged under protocol number CAAE 92928518.3.0000.5149. The study followed the Consolidated Standards of Reporting Trials (CONSORT) and was registered on the Brazilian registry of clinical trials (ReBEC platform) under number RBR-22j8728. All participants provided voluntary written consent and agreed with all the study procedures. Participants were also told they could opt out of the study at any time.

### Study design

The study is a quasi-experimental clinical trial with a blind before and after comparison, in addition to nine months of follow-up after the end of the intervention.

The effects of GVS were tested in subjects with HAM. The balance assessment was done before, on the 6th and 12th week after the beginning of the intervention. During the nine months of follow-up, the patients did not receive stimulation and were submitted to balance assessment after the ninth month without stimulation.

### Participants

The study included patients with HAM recruited from an outpatient clinic specialized in HTLV-1 infection (Table 2). For the diagnosis of HAM, the criteria proposed by the World Health Organization (WHO), adapted by Castro-Costa et al. (2006), classified according to the expanded functional disability scale (EDSS) (Kurtzke, 1983) and OSAME Revised Motor Disability Scale (OMDS) (Osame, 1990), showing a rating of at least one in one of the scales (Castro-Costa et al., 2006; Kurtzke, 1983). The exclusion criteria were recurrent episodes of vertigo or a single episode of vertigo lasting more than 30 min or history of previously diagnosed vestibulopathy; individuals with a history of myelitis or stroke or other neurological disease that can affect balance; individuals with immobility (use of a wheelchair or walking aid); people with a pacemaker or any other electronic device that can undergo changes due to GVS; individuals with positive serology for HTLV-2, HIV, syphilis.

The patients were evaluated through a clinical interview and then submitted to an otoneurologic examination.

### Galvanic vestibular stimulation

**Figure 1:**
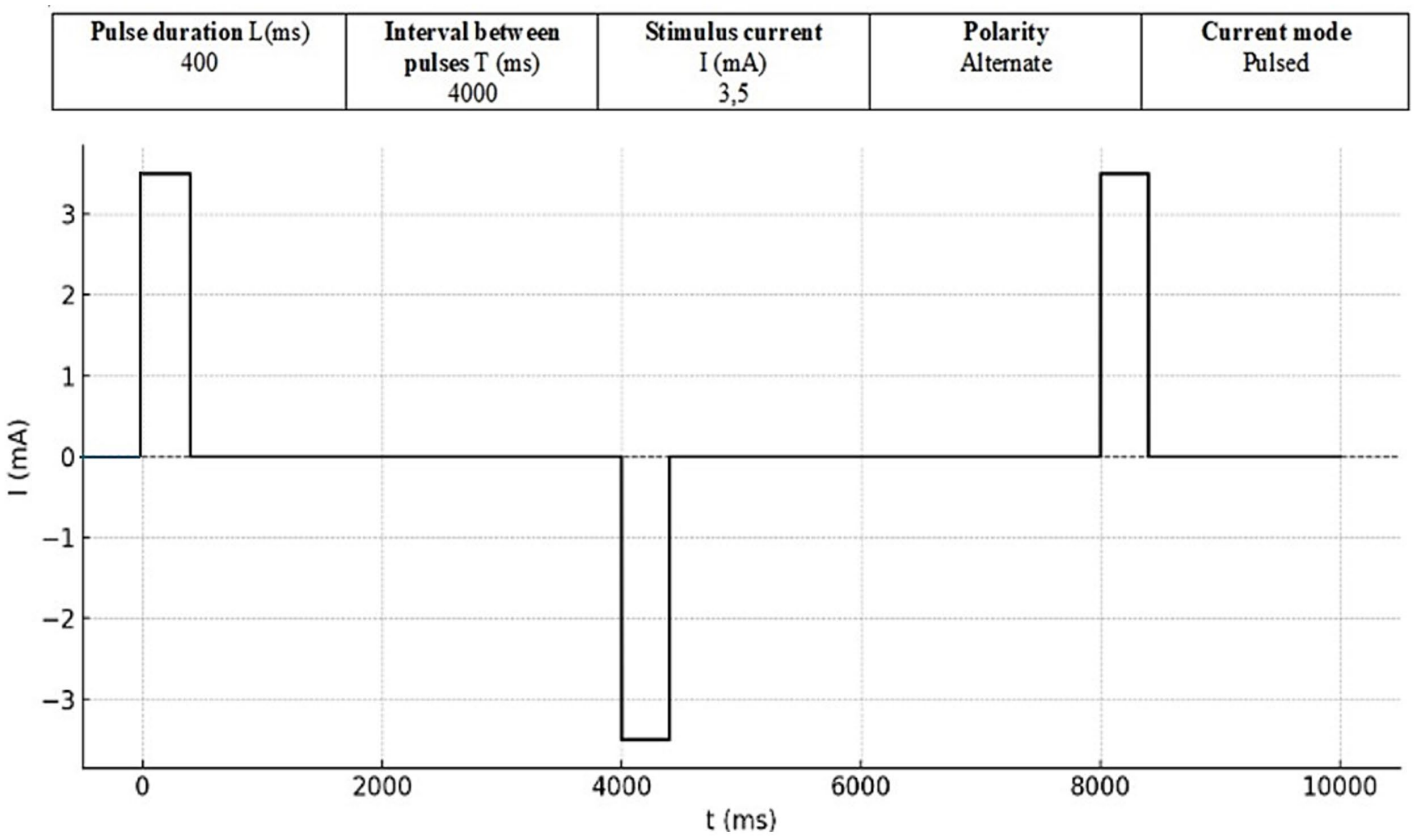
Stimulus format.

**Figure 2:**
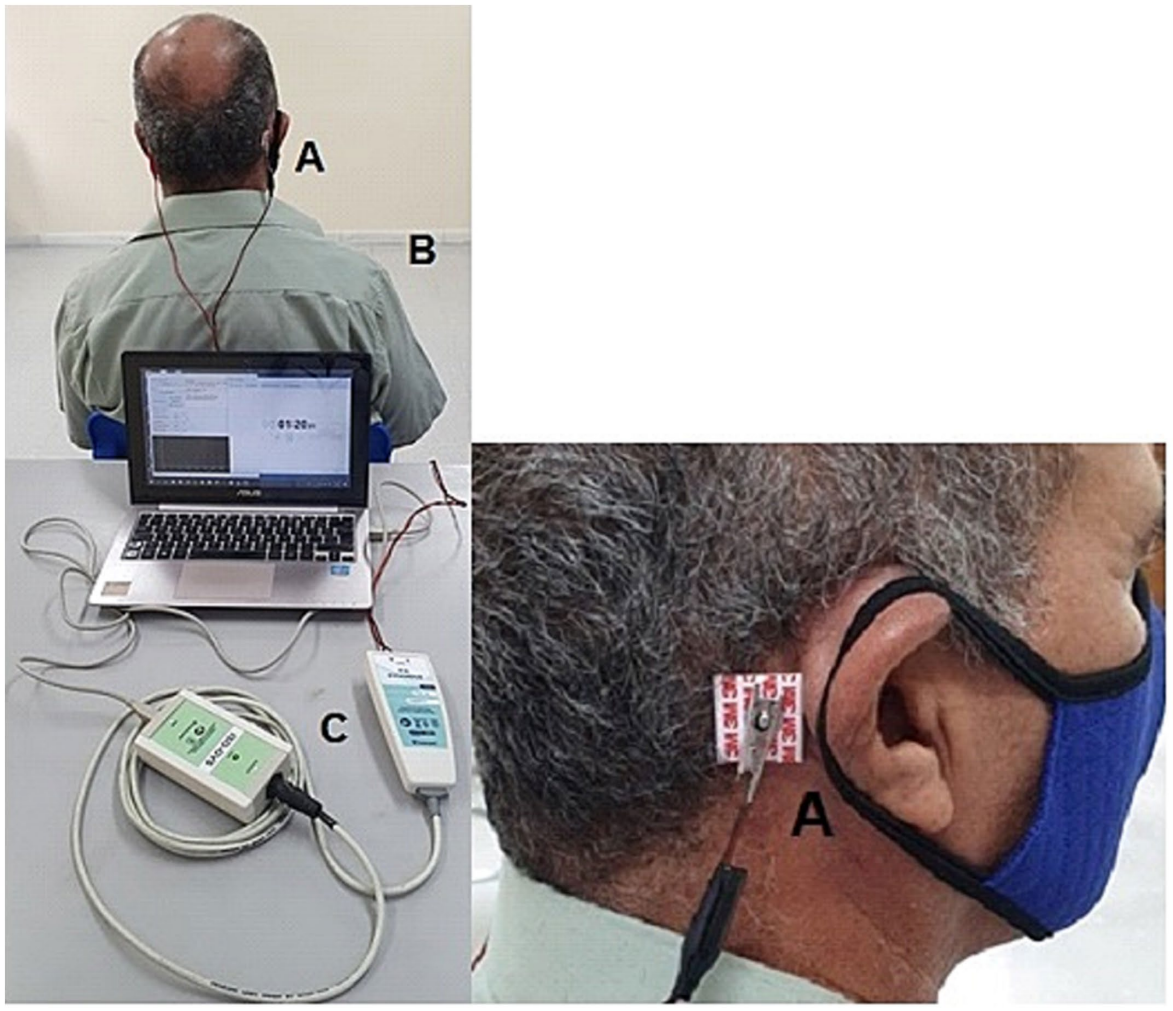
Patient undergoing the electrical (galvanic) vestibular stimulus with the electrodes on both mastoid bones. **(A)** Electrodes on the mastoids; **(B)** patient submitted to GVS must be seated; **(C)** GVS equipment connected to the computer.

The intervention was the GVS. The equipment (Contronic^®^, Brazil) consisted of an electrical stimulator that was programmed to release a uniphasic, rectangular pulse with variable current intensity and a duration of 400 milliseconds (ms). Disposable, self-adhesive surface electrodes were attached to both mastoid processes, offering binaural and bipolar current in which cathode and anode alternated every 4 s. The duration of each pulse was 400 ms, with an interval of 4 s (Figure 1). During the intervention, the individual was instructed to sit in a chair, remove shoes and objects that could be good conductors of electricity and keep their eyes closed (Figure 2).

GVS was applied during 12 consecutive weeks with one session per week, 3 series of stimulation in each session. The intervention started with the current intensity of 1.0 milliamps (mA) and it was progressively increased until reaching a maximum current of 3.5 mA. In the first and second weeks, each series was progressively increased by 0.5 mA until reaching 2.5 mA. In the third week, the current intensity was not changed. In the fourth week, the intensity was increased by 0.5 mA and the protocol reached 3.0 mA, which was maintained in the fifth week. In the sixth week, there was an increase of 0.5 mA and the current of 3.5 mA was reached. From the seventh to the twelfth week, ascending current series between 3.0 and 3.5 mA were used. Regarding the tolerance of the current intensity, the sensation threshold reported by the participants was between 3.0 and 3.5 mA. All participants reported minimal discomfort, such as slight tingling in the mastoid region and slight head shaking. Considering that 3.5 mA has been shown to be sufficient to stimulate the central vestibular system, the intensity of the protocol was maintained up to this intensity level aiming at safety and tolerance to the stimulus (Vailleau et al., 2011; Čobeljić et al., 2018; Dilda et al., 2012).

The definition of the time of stimulation was based on a published protocol using GVS for balance rehabilitation (Carmona et al., 2011; Čobeljić et al., 2018). The electrical stimulation time was progressively increased from 9 min in the first session to 18 min in the second session and maintained at 30 min from the third session onwards.

### Balance assessment

Balance assessment was based on postural subjective and objective tests that were done before the intervention, after the 6th and the 12th session of GVS and after 9 months of following-up without stimulation. The tests performed were: timed up and go test (TUG), Berg balance scale (BBS) and static posturography (POST) (Contronic, Brazil).

TUG aims to assess mobility and balance. The test quantifies functional mobility in seconds through the time the individual gets up from a chair, walks 3 meters, turns around, returns to the chair and sits down again (Podsiadlo and Richardson, 1991). For TUG in HAM patients, the considered altered result was a time greater than 10 s to complete the test (Podsiadlo and Richardson, 1991).

BBS evaluates the perception of functional independence to perform activities of daily living that require body balance. The scale assesses balance at 14 items; each has an ordinal scale of five alternatives ranging from 0 to 4 points. Points are based on the time that a position can be maintained, at the distance the upper limb can reach in front of the body and in time to complete the task (Miyamoto et al., 2004). For BBS, values lower than 50 points indicate a greater risk of falls (Miyamoto et al., 2004).

POST assesses body balance in terms of the influence of visual, somatosensory and vestibular stimuli. The participants were evaluated regarding anteroposterior (AP) and mediolateral (ML) plane displacement concerning balance movement and its alterations through stimuli that were measured at an interval of not less than 30 s and not more than one minute, giving preference to the interval of 45 s. All POST tests were performed with the patient standing with their feet on the platform, and the equipment software calculated all the data automatically.

The POST analysis was based on the Stability Limit (SL) and on the Sensory Organization Test (SOT) under the following conditions: (1) eyes open on a stable surface (EOS); (2) eyes closed on a stable surface (ECS); (3) eyes open on an unstable surface (EOU); (4) eyes closed on an unstable surface (ECU); (5) visual tunnel effect on a stable surface (TS); (6) head 30° up with eyes closed (HUEC); (7) head 30° down with eyes closed (HDEC).

The SL was evaluated by asking the participant to perform a maximum body displacement in anteroposterior and lateral directions using only the ankle strategy, that is, without using trunk and foot movement strategies to control balance. Subsequently, SOT was measured under seven conditions.

The SOT provides quantitative data regarding the influence of vision, proprioception and labyrinth in the control of body balance (Bittar, 2007; Oda and Ganança, 2015). The sequence of SOT conditions was the same for all participants and followed the order of starting with the easiest condition to maintain balance and progressively moving to the more challenging ones.

For each condition, the measured parameters were stability limit area (SLA) and confidence ellipse (CE). SLA indicates the maximum postural displacement the patient can reach on the ML and AP axes.

CE is a graphic technique that delimits the area, measured in square millimeters (mm^2^), in a scatter plot based on points and paths obtained in SLA. In the device software, the ellipse will contain at least 95% of the center of pressure points collected throughout the test. The points corresponding to the center of pressure measured continuously on the POST platform were stored in two vectors, AP and ML. The mean value of each vector was calculated. Then, the matrix was calculated. Subsequently, the eigenvalues and eigenvectors of the covariance matrix were computed, determining the directions and intensities of the ellipse axes. The applied method was implemented in the mathematical software Scilab and converted to code in C++ language for incorporation into the POST platform software (Johnson and Wichern, 2007; Charles, 1991; Systemes, 2017; Embarcadero Technologies, 2010). Finally, the calculation method was validated by inserting AP and ML vectors with predetermined values. The system composed of platform and software was evaluated and approved by INMETRO (Brazilian Institute of Metrology: Certificate of Conformity—UL-BR 17.0323—May 2, 2017) and ANVISA (National Health Surveillance Agency: Process 25351.263332-2017-00—Clearance 80,384,070,006).

### Statistical analysis of data

Statistical analysis was performed using the *Statistical Package for Social Sciences* (SPSS), version 20.0. A paired comparative analysis of the individuals before and after treatment with GVS was performed. The analyzed variables were SL, CE and SLA in the POST, the time required in TUG, the total score and the score for each domain in BBS. The normality of the samples was assessed using the Kolmogorov–Smirnov and Shapiro–Wilk tests. Since the distribution of the variables was not normal, the comparison between the groups was performed using nonparametric tests. The intragroup comparison before and after the intervention was performed using the Friedman test. For multiple comparisons, Friedman’s two-way ANOVA was used. For the comparative analysis between the visual sensory organization and the time of galvanic vestibular stimulation, the two-way ANOVA test was used. The adopted level of significance was 5% (*p* ≤ 0.05).

## Results

Regarding complaints related to the stimulation, all patients reported a slight head nodding and a tingling sensation or small shock in both mastoids within the current intensity of 3.5 mA. No other side effect was observed (see Table 1).

**Table 1:** Protocol of galvanic vestibular stimulation – 1 session per week for 12 weeks.

Series	Sessions							
	1	2	3	4	5	6	7	8–12
1	[1.0/1/3]	[2.0/2/3]	[2.0/2/5]	[2.5/2/5]	[2.5/2/5]	[2.5/2/5]	[3.0/2/5]	[3.0/2/5]
2	[1.5/1/3]	[2.5/2/3]	[2.5/2/5]	[3.0/2/5]	[3.0/2/5]	[3.0/2/5]	[3.0/2/5]	[3.5/2/5]
3	[2.0/1/3]	[2.5/2/3]	[2.5/2/5]	[3.0/2/5]	[3.0/2/5]	[3.5/2/5]	[3.5/2/5]	[3.5/2/5]

Current in milliamp/stimulus duration time in minutes/number of stimulus repetitions. Each session consisted of 3 series of stimuli.

Table 2 presents the characteristics of the participants regarding age, sex, duration of disease and postural instability. The sample was homogeneous for these variables (*p* > 0.05).

**Table 2:** General characteristics of 20 patients with HTLV-1 associated myelopathy.

Gender	*n*	Age (years)	DT (years)	PI (years)
Female	12 [60]	77.5 (75.0/81.0)	9.5 (8.0/10.5)	5.5 (5.0/6.0)
Male	8 [40]	80.5 (78.0/81.5)	9.0 (9.0/10.0)	5.5 (5.0/6.5)

*n* = number of participants, absolute number (percentage), DT = disease time, PI = postural instability time, Data are expressed as median (1°/3° percentiles).

Table 3 presents the comparison of TGU and BBS. After the intervention, the reduction in time to execute TUG and the increase in BBS score indicated an improvement in the walking speed and in the patient’s ability to safely balance during the predetermined BBS tasks.

**Table 3:** Timed up and go test and Berg balance scale in 20 patients with HTLV-1-associated myelopathy with the comparison between the different moments of the intervention.

Tests	T0	T1	T2	T3	*p*-value*	Comparison groups**
TGU	28.00 (26.25/29.00)	23.00 (21.25/24.00)	18.00 (17.00/19.00)	24.00 (23.00/25.00)	<0.001	T0 X T1^#^ T0 X T2^#^ T0 X T3^#^ T1 X T2^#^ T1 X T3 T2 X T3^#^
BBS	29.00 (28.00/31.00)	34.50 (33.00/36.00)	41.00 (39.00/41.75)	33.50 (32.00/35.00)	<0.001	T0 X T1^#^ T0 X T2^#^ T0 X T3 T1 X T2 T1 X T3 T2 X T3^#^

T0 = before Galvanic Vestibular Stimulation, T1 = after the 6th session of Galvanic Vestibular Stimulation, T2 = after the 12th session of Galvanic Vestibular Stimulation, T3 = 9 months follow-up without Galvanic Vestibular Stimulation, TGU = Timed up and go test, BBS = Berg balance scale, median (1° quartile/3° quartile). * Friedman Test/Friedman’s two-way ANOVA (*p* ≤ 0.05). **Bonferroni Test. # (*p* ≤ 0.05).

Table 4 presents the patients’ performance in the POST, and an improvement in the SOT was observed for all conditions tested after the 12th stimulation session. However, after 9 months without stimulation, the gain was maintained for conditions considered less challenging for balance (EOS, ECS, and TS) and lost for conditions requiring better balance control.

**Table 4:** Static posturography of 20 patients with HTLV-1 associated myelopathy in the seven tested conditions with the comparison of the performance between the different moments of intervention.

POST	T0	T1	T2	T3	*p* – value*	Comparison groups**
ECS	4.14 (1.99/7.13)	2.72 (1.96/4.00)	1.14 (1.08/1.20)	1.74 (1.33/2.77)	<0.001	T0 X T1 T0 X T2^#^ T0 X T3^#^ T1 X T2^#^ T1 X T3 T2 X T3
EOS	2.56 (2.20/3.29)	1.42 (1.21/1.66)	1.99 (1.82/2.20)	1.78 (1.61/2.18)	<0.001	T0 X T1^#^ T0 X T2^#^ T0 X T3^#^ T1 X T2^#^ T1 X T3^#^ T2 X T3
EOU	1.90 (1.62/2.89)	1.87 (1.75/2.18)	1.10 (1.00/1.17)	1.56 (1.38/2.20)	<0.001	T0 X T1 T0 X T2^#^ T0 X T3^#^ T1 X T2^#^ T1 X T3 T2 X T3^#^
ECU	2.15 (1.68/3.27)	1.87 (1.75/2.18)	1.17 (1.04/1.50)	2.15 (1.68/3.27)	<0.001	T0 X T1 T0 X T2^#^ T0 X T3 T1 X T2^#^ T1 X T3 T2 X T3^#^
TS	5.75 (3.84/6.89)	3.19 (2.18/4.11)	1.53 (1.23/2.54)	3.07 (2.03/4.16)	<0.001	T0 X T1^#^ T0 X T2^#^ T0 X T3^#^ T1 X T2^#^ T1 X T3 T2 X T3^#^
HUEC	1.87 (1.75/2.18)	1.54 (1.42/1.67)	1.14 (1.08/1.20)	1.78 (1.61/2.18)	<0.001	T0 X T1^#^ T0 X T2^#^ T0 X T3 T1 X T2^#^ T1 X T3 T2 X T3^#^
HDEC	1.78 (1.61/2.18)	1.42 (1.21/1.66)	1.10 (1.00/1.17)	1.87 (1.75/2.18)	<0.001	T0 X T1^#^ T0 X T2^#^ T0 X T3 T1 X T2^#^ T1 X T3^#^ T2 X T3^#^

POST = static posturography, T0 = before Galvanic Vestibular Stimulation (GVS), T1 = after the 6th session of GVS, T2 = after the 12th session of GVS, T3 = 9 months without GVS, EOS = eyes open on a stable surface; ECS = eyes closed on a stable surface; EOU = eyes open on an unstable surface; ECU = eyes closed on an unstable surface; TS = tunnel visual effect on a stable surface; HUEC = head 30° up eyes closed; HDEC = head 30° down with eyes closed, median (1° quartile/3° quartile) for 95% confidence area of the ellipse in square millimeters. * Friedman Test/Friedman’s two-way ANOVA (*p* ≤ 0.05). **Bonferroni Test. # (*p* ≤ 0.05).

**Figure 3:**
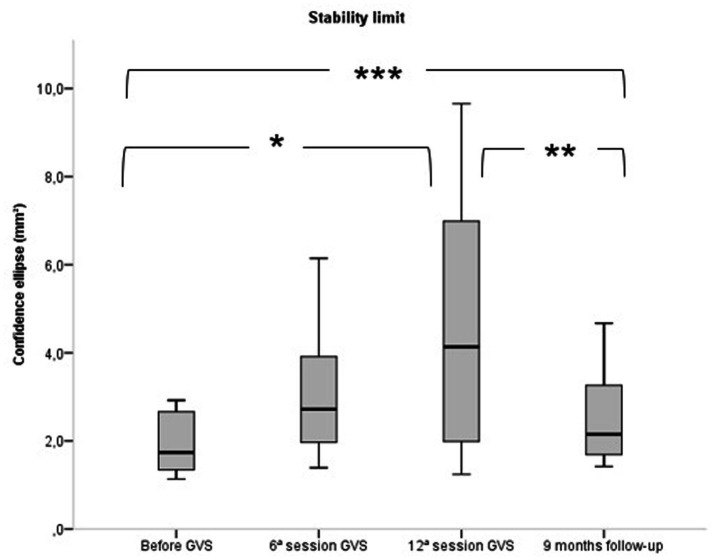
Comparison of stability limit in static posturography of 20 patients with HTLV-1 associated myelopathy before, after the 6th session and 12th session of Galvanic Vestibular Stimulation and after the 9th month of follow-up without galvanic vestibular stimulation. GVS, Galvanic Vestibular Stimulation. *p ≤ 0.05. Friedman Test / Friedman’s two-way ANOVA (*p* ≤ 0.05). Bonferroni Test.

**Figure 4:**
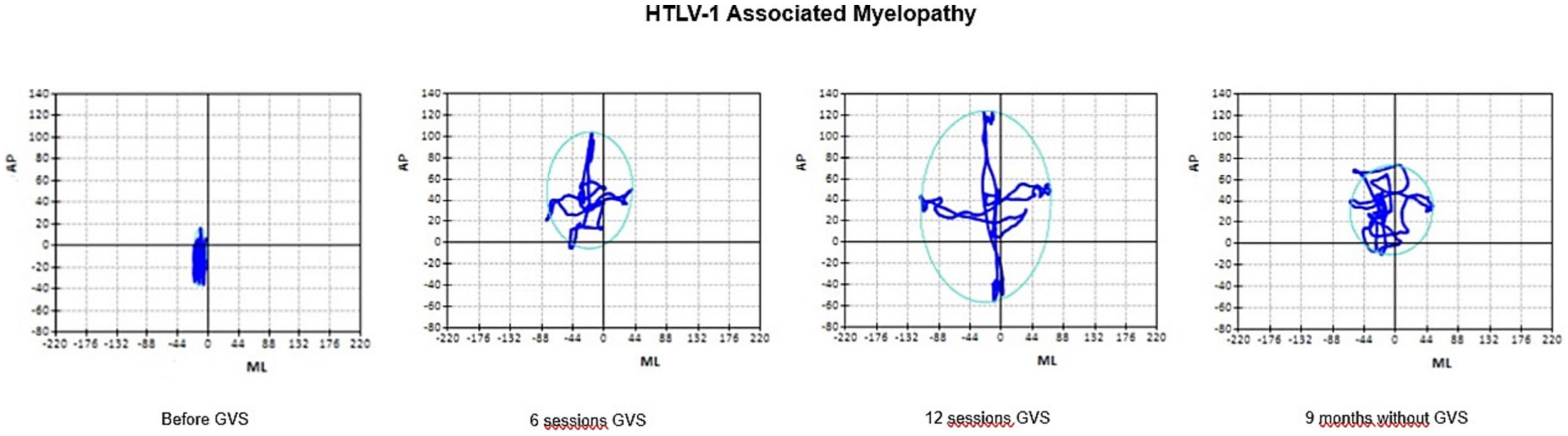
Average performance of 20 patients with HTLV-1 Associated Myelopathy in POST in relation confidence ellipse, before GVS, after the 6th and the 12th session of GVS and after 9 months follow-up without GVS. AP, displacement anteroposterior displacement; ML, displacement mediolateral displacement; GVS, Galvanic Vestibular Stimulation. Data are expressed as median.

Regarding the stability limit, Figure 3 shows that it improved at the end of the intervention (12th session) but went back to baseline after 9 months without stimulation. The same result was found in CE, shown in Figure 4, in which the scatter plot represents the SLA of the 20 participants. In fact, all tests showed a consistent improvement in balance after the end of the intervention, followed by the loss of the gain with the interruption of GVS.

## Discussion

GVS is a safe stimulation method of the vestibular system (Nguyen et al., 2022) and can be used for testing the integrity of the vestibulospinal tract (Kwan et al., 2019; Tohyama et al., 2021; Wuehr et al., 2023; Carmona et al., 2011) and for postural rehabilitation.

In postural rehabilitation protocols using GVS, the polarity of the current varies according to the postural effect. In postural instabilities caused by neurological diseases, the GVS configuration is programmed to reverse the polarity several times during the rehabilitation session (Carmona et al., 2011). In postural instabilities caused by peripheral diseases, such as unilateral vestibulopathy, GVS configuration is set up to keep the cathode on the compromised labyrinth throughout the rehabilitation session without polarity reversal, so that the impaired vestibulospinal reflex is continuously stimulated (Nguyen et al., 2022; Silva et al., 2019). Considering the patients in the present study, the cathode/anode polarity was reversed during GVS, aiming at similar electrical current in both sides.

Because the membrane potential of both hair cells and afferent fibers is sensitive to electrical currents, GVS could either trigger action potentials in vestibular afferents directly or indirectly through hair cell depolarization and increased transmitter release or recruit both cellular substrates. The partial reduction of GVS-induced vestibular afferent discharge modulation, when the hair cell–afferent synaptic transmission was blocked, indicates that, under control conditions, both hair cells and afferent fibers are recruited by galvanic vestibular stimulation even though a direct activation of vestibular afferents predominated at higher stimulus intensities (Gensberger et al., 2016).

Sinusoidal galvanic stimulation of semicircular canals provokes a modulation of neuronal activity in all VOR elements. The GVS site dependency of response magnitudes and matching spatiotemporal profiles during GVS and rotation demonstrate that applied currents activate canal-specific circuits and imitate natural head movements over a broad range of frequencies and amplitudes. The partially reduced GVS-evoked vestibular afferent discharge following a block of the glutamatergic transmission indicates that galvanic currents recruit both hair cells and vestibular nerve afferent fibers (Gensberger et al., 2016).

The present study showed an improvement in body balance as demonstrated by the reduction in the time to execute TUG and the increase in BBS scores (Table 3). GVS stimulates neuronal connections, which may have favored partial recovery of the vestibular function. However, the gain was lost after the interruption of the stimulation. This finding is crucial since HAM presents a progressive worse that is part of the physiopathology of the disease. Perhaps, for peripheral vestibular diseases, an effective neuromodulation occurs considering the normal CNS. Studies about the follow-up of peripheral vestibulopathies regarding the maintenance of the gain after the interruption of GVS are not available so far.

The posturography results on a force platform demonstrated that GVS promoted a gain in functionality, with an increase in the confidence ellipse area for the stability limit and a reduction in the area of the confidence ellipse in the other tests (Figures 3, 4). The posturography also showed that with the interruption of the GVS, there was a reduction in the stability limit (Figures 3, 4). Possibly, GVS was able to stimulate synapses of the remaining neuronal tissue while electrical current was applied, but permanent recovery could not occur, as HAM leads to progressive upper motor neuron loss.

The stability limit corresponds to the area of movement that an individual can move comfortably in all directions (anterior, posterior, medial and lateral) without falling. Postural instability reduces the stability limit, as observed in the stability limit map before the start of the intervention (Figure 4).

The reduction of the CE after the intervention indicated a balance improvement. Before GVS, regarding the eyes-open condition, for both the stable and unstable surfaces, the CE was bigger comparing to the eyes-closed conditions. The visual information explains this result. Interestingly, after GVS, in the conditions without visual information, either in stable or unstable conditions, an improved performance was observed that approached to that observed in the eyes-open conditions. This finding seems to indicate an improvement of the vestibular loop related to balance control that led to balance gain even without visual information.

Postural control and maintenance of body balance depend on the vestibular system and sensory information coming from the somatosensory, visual and vestibular systems, integrated in the brain stem. All together influences the effective motor control related to balance. Information from the peripheral visual field appears to be more important for postural control than focal information. Visual feedback allows for less variability in the displacement of the center of pressure in long-term orthostatic posture. The visual system also contributes to maintaining natural balance within the limits of the support base, informing how to maintain the alignment of the head and trunk when the center of mass is disturbed by the translation of the support base (Chen et al., 2021).

The visual and somatosensory systems are primarily more sensitive to low-frequency stimulation, such as postural sway, which is less than 0.5 Hz, and gait, which is less than 1.0 Hz. Another important aspect of the visual contribution is the threshold for perception of postural sway at low movement speeds, which is greater than of the proprioceptive system. At higher speeds, on the other hand, both systems present similar perception (Chen et al., 2021; Mitsutake et al., 2022).

Regarding the somatosensory system, it has been demonstrated that receptors in the feet can interfere with the threshold of spinal neurons, which interact with vestibular, visual and proprioceptive information from the neck. It is also recognized that these mechanoreceptors are capable of localizing and detecting small changes in pressure on the soles of the feet to react to high frequency. Receptors in the feet, legs, and trunk are important for body control, especially under conditions where the individual remains in contact with a large, rigid, stable surface (Chen et al., 2021; Tohyama et al., 2021). After GVS, participants’ performance improved in the visual tunnel effect (TS) condition, which is related to connections related to the somatosensory system. Somatosensory information not only informs the nervous system about the qualities of the support surface, but also about the force that the body exerts on these surfaces, the position and speed of all body segments, their contact with external objects, including the ground, and the orientation of gravity (Chen et al., 2021).

The results of the present study showed that GVS had an effect on the vestibular system. The conditions of controlled visual and somatosensory information deficit allowed to see the better vestibulospinal reflex after GVS. After the end of the intervention, the improved balance was seen even in the condition of standing on an unstable surface, without vision or external support (Hlavacka et al., 1999; Welgampola and Colebatch, 2001). The anatomical separation of the sensory systems involved in postural control and the significant degradation of sensory information when we close our eyes or stand on soft or smooth surfaces suggest that the nervous system has the ability to discreetly change the main source of sensory information (Chen et al., 2021).

Galvanic vestibular stimulation is a type of neuromodulation that modifies the complex patterns of transmission of electrical and neurochemical signals in the CNS. It acts on the neural communication network to balance brain waves and release neurotransmitters. Neuronal stimulation provides faster synaptic communication and, therefore, over time, there will be greater functionality in this network, improving the synaptic plasticity of the involved region. The neuroplasticity provided by neuromodulation is capable of reorganizing neural connections and, consequently, reestablishing the balance of neuronal activity related to postural instability (Hlavacka et al., 1999; Welgampola and Colebatch, 2001).

Regarding the intensity of the electric current, the GVS protocol is variable. Some authors believe that the beginning of the intervention should be defined by the lowest current, the lowest stimulus time and the lowest number of repetitions, which are progressively increased throughout the treatment according to the individual’s tolerance (Silva et al., 2020; Wuehr et al., 2023). Other authors suggest a fixed protocol without any variation in the current intensity and stimulation time (Kwan et al., 2019). The chosen protocol in the present study was focused on reducing the patient’s anxiety in facing an intervention modality that involves electrical stimulus. We started with the lowest current and the shortest stimulus time, which was increased throughout the sessions. The advantage was the reduction of stress during the intervention since sensitivity to this type of stimulus may vary among individuals. This protocol was very well tolerated and showed positive gains regarding postural balance.

GVS has been shown to be a safe technique that improves body balance in the short term during the stimulation period, but the effect is lost after interruption. Thus, this neuromodulation strategy could be considered in the postural balance rehabilitation approach for home use. On the other hand, broader evidence in neurorehabilitation suggests that pairing electrical stimulation with active rehabilitation tasks can lead to enhanced outcomes. However, this strategy is not applied to patients with chronic fatigue and depression, which characterizes the profile of HAM patients. Therefore, the rationale for using a GVS protocol, where the patient remains seated without concurrent physical rehabilitation, is the primary approach for individuals unable to follow a protocol that requires physical involvement. For this specific group of patients, who cannot undergo active physical rehabilitation due to the inherent limitations of their condition, a passive rehabilitation program can be an excellent intervention strategy. As a next step, depending on improvements from the passive rehabilitation, a structured physical exercise program can be introduced.

The study sample was small, and there was no control group that received a sham stimulus. Another limitation is that it is not possible to definitively attribute the observed changes in posturography to GVS, as they may be influenced by participants’ prior exposure to the testing conditions, a potential “learning effect.” Despite these limitations, positive effects on postural balance were observed, considering the posturographic data alongside an improvement in walking speed. These pre- and post-intervention measurements were based on objective data that were analyzed blindly. While it is impossible to completely rule out bias due to the absence of a sham group, the present study provides evidence that the improvement in postural balance was likely caused by GVS rather than a learning effect or placebo response.

Regarding follow-up, longitudinal studies are prone to participant attrition. In this study, there was no participant attrition. All participants were tested at baseline, again during the trial, and finally 9 months after the end of the intervention. Therefore, unbiased measurements combined with follow-up of all participants increase the quality of the results, despite the limitation of not having a sham group. Ultimately, the current results warrant further attention in future studies with sham control groups to confirm and strengthen our conclusions.

## Conclusion

GVS provided a progressive improvement in balance, comparing the results before, during and after the intervention. Thus, GVS was shown to help improve the body balance of patients with HAM. The gain was not maintained in the long term after the interruption of the stimulation.

## Data Availability

The datasets presented in this study can be found in online repositories. The names of the repository/repositories and accession number(s) can be found in the article/Supplementary material.
