# Supplementary material for: Galvanic vestibular stimulation for the postural rehabilitation of HTLV-1-associated myelopathy
Source: Front Hum Neurosci. 2024 Dec 19;18:1507559. doi: 10.3389/fnhum.2024.1507559 (PMC11693613; doi:10.3389/fnhum.2024.1507559)
Supplement: Supplementary file 1 [file Data_Sheet_1.pdf]

DATE ___/___/___

IDENTIFICATION

ID SEARCH ________________________ DATE OF BIRTH ______/______/______ AGE___________ gender __________________

NAME ____

ADDRESS ____

____

ZIP CODE ____ TELEPHONE ______________________ / ____________________

EMAIL ____

CLINICAL EVOLUTION

____

____

____

____

____

____

____

____

____

PHYSICAL EXAM

Weight: |____|____|____|, |____| Kg Height/Stature: |____|____|____|, |____| cm Axillary temperature: |____|____|,|____| °C

BLOOD PRESSURE: _____ _____ _____ / _____ _____ _____ mmHg HEART RATE: |_____|_____|_____| b.p.m

RESPIRATORY FREQUENCY.: |_____|_____| i.p.m.

PRE-EXISTING MEDICAL CONDITIONS:

____

____

____

____

____

____

____

MEDICATIONS IN USE:

____
____
____
____
____
____
____

SPEECH THERAPY ASSESSMENT: AUDITORY AND VESTIBULAR SYMPTOMS

____
____
____
____
____

TIMED UP AND GO TEST

Time ( ) less than 10 seconds ( ) greater than 10 seconds

BERG BALANCE SCALE

Punctuation ( ) less than or equal to 49 ( ) greater than 49
