# Supplementary material for: Galvanic vestibular stimulation for the postural rehabilitation of HTLV-1-associated myelopathy
Source: Front Hum Neurosci. 2024 Dec 19;18:1507559. doi: 10.3389/fnhum.2024.1507559 (PMC11693613; doi:10.3389/fnhum.2024.1507559)
Supplement: Supplementary file 3 [file Data_Sheet_3.pdf]

S2 Table. Descriptive variables of patients with HTLV-1-associated myelopathy: age, disease time, postural instability time, disability scales (EDSS and OMDS), Timed up and go test and Berg balance scale

Variables		HTLV-1-associated myelopathy
**Age**	Mean	78.35
	Standard deviation	3.72
	Median	79.50
	Minimum	71.00
	Maximum	84.00
**Disease time**	Mean	9.35
	Standard deviation	1.35
	Median	9.00
	Minimum	7.00
	Maximum	12.00
**Postural instability time**	Mean	5.45
	Standard deviation	1.19
	Median	5.50
	Minimum	3.00
	Maximum	7.00
**EDSS**	Mean	0.00
	Standard deviation	0.00
	Median	0.00
	Minimum	0.00
	Maximum	0.00
**OMDS**	Mean	0.00
	Standard deviation	0.00
	Median	0.00
	Minimum	0.00
	Maximum	0.00
**TUG Before GVS**	Mean	27.75
	Standard deviation	1.51
	Median	28.00
	Minimum	25.00
	Maximum	30.00
**TUG after 6th session GVS**	Mean	22.95
	Standard deviation	1.66
	Median	23.00
	Minimum	20.00
	Maximum	26.00
**TUG after 12th session GVS**	Mean	18.05
	Standard deviation	1.50
	Median	18.00
	Minimum	16.00
	Maximum	21.00

**TUG 9 months follow-up**	Mean	23.85
	Standard deviation	1.18
	Median	24.00
	Minimum	22.00
	Maximum	26.00
**BBS Before GVS**	Mean	29.30
	Standard deviation	1.69
	Median	29.00
	Minimum	27.00
	Maximum	32.00
**BBS after 6th session GVS**	Mean	34.40
	Standard deviation	1.39
	Median	34.50
	Minimum	32.00
	Maximum	36.00
**BBS after 12th session GVS**	Mean	40.50
	Standard deviation	1.38
	Median	41.00
	Minimum	38.00
	Maximum	43.00
**BBS 9 months follow-up**	Mean	33.40
	Standard deviation	1.39
	Median	33.50
	Minimum	31.00
	Maximum	35.00
