# Supplementary material for: Galvanic vestibular stimulation for the postural rehabilitation of HTLV-1-associated myelopathy
Source: Front Hum Neurosci. 2024 Dec 19;18:1507559. doi: 10.3389/fnhum.2024.1507559 (PMC11693613; doi:10.3389/fnhum.2024.1507559)
Supplement: Supplementary file 4 [file Data_Sheet_4.pdf]

S3 Table. Descriptive variables the performance of patients with HTLV-1-associated myelopathy in Posturography before, after the 6th session and after the 12th session of GVS and 9 months of follow-up without GVS

Posturography		HTLV-1-associated myelopathy			
		Before	6th session GVS	12th session GVS	Follow-up
Stability limit	Mean	2.43	3.18	4.59	2.63
	Standard deviation	1.69	1.67	2.77	1.06
	Median	1.74	2.72	4.14	2.15
	Minimum	1.14	1.39	1.24	1.42
	Maximum	6.53	7.53	9.65	4.67
Eyes open on a stable surface	Mean	2.81	1.43	2.02	1.86
	Standard deviation	0.73	0.28	0.29	0.33
	Median	2.56	1.42	1.99	1.78
	Minimum	1.83	1.01	1.64	1.40
	Maximum	4.42	2.01	2.90	2.61
Eyes closed on a stable surface	Mean	4.59	3.18	1.13	2.43
	Standard deviation	2.77	1.67	0.80	1.69
	Median	4.14	2.72	1.14	1.74
	Minimum	1.24	1.39	1.00	1.14
	Maximum	9.65	7.53	1.27	6.53
Eyes open on an unstable surface	Mean	2.28	1.90	1.12	1.85
	Standard deviation	0.95	0.28	0.11	0.68
	Median	1.90	1.87	1.10	1.56
	Minimum	1.37	1.17	1.00	1.20
	Maximum	4.33	2.30	1.47	3.23
Eyes closed on an unstable surface	Mean	2.63	1.90	1.35	2.63
	Standard deviation	1.06	0.28	0.44	1.06
	Median	2.15	1.87	1.17	2.15
	Minimum	1.42	1.17	1.00	1.42
	Maximum	4.67	2.29	2.35	4.67
Tunnel visual effect on a stable surface	Mean	5.42	3.31	1.84	3.36
	Standard deviation	1.95	1.35	0.70	1.63
	Median	5.75	3.19	1.53	3.07
	Minimum	1.94	1.68	1.08	1.61
	Maximum	8.94	6.91	3.21	7.53
Head 30º up with eyes closed	Mean	1.90	1.55	1.13	1.86
	Standard deviation	0.28	0.18	0.80	0.33
	Median	1.87	1.54	1.14	1.78
	Minimum	1.17	1.20	1.00	1.40
	Maximum	2.29	1.89	1.27	2.61
Head 30º down with eyes closed	Mean	1.86	1.43	1.12	1.90
	Standard deviation	0.33	0.28	0.11	0.28
	Median	1.78	1.42	1.10	1.87
	Minimum	1.40	1.01	1.00	1.17
	Maximum	2.61	2.01	1.47	2.29
